# The Man with One Paper

**Published:** 1901-10

**Authors:** 


### Editorial.

### THE MAN WITH ONE PAPER.

The man with "one talent," if this talent or idea be used to the advantage and instruction of his fellows, is worthy of all admiration. All are not gifted alike, some are capable of doing many things equally well, while others must confine themselves to certain lines of work, and can do nothing beyond these worthy of notice. The cultivation of the one original idea has more than once not only made the man who possessed it financially comfortable, but has enriched the world. In fact, this may be said to be more universally true than the results obtained by those whose minds cover many things. The Edisons are a rarity, but Stephenson, Fitch, Fulton, Morse, Bell, etc., have been the representative types of progress in all ages and in all countries.

While this is true, the man with one idea in dentistry is apt to become burdensome if he thinks it necessary to duplicate this and reduplicate it just as long as he can find editors willing to publish. Readers of journals are familiar with this type, and editors have a feeling akin to horror when they have sent them the same old story, variously disguised under changed headings.

It is recognized that a single publication of an original paper rarely makes an impression. This is well understood by advertisers. They do not count on one or a dozen appearances of an advertisement to effect anything. They understand fully that it is only through constant repetitions that the mind gradually absorbs the idea sought to be conveyed, and that eventually the recipient is drawn irresistibly into purchasing the product. "See that Hump?" and "Uneeda Biscuit" simply typify the fortunes made by thus playing upon a single string.

The modest man who, having spent weary days and nights evolving a truth in science, gives it out in a paper, and, having done this, feels, in a certain sense, relieved of all responsibility and cares very little whether the world receives his message or permits it to die unnoticed, yet sometimes finds that he is taken at his own valuation, and the world passes him by to glorify inferior men. The history of scientific research is full of these forgotten workers. When Cohnheim electrified the medical world by his discovery of the migration of the leucocytes of the blood, and aroused thereby an almost endless antagonism from the advocates of the earlier theory of Virchow of cell proliferation, the work of Waller, of England, and his contemporaries was entirely forgotten, and yet the results achieved by Waller were, in all respects, equal to that of the German investigator.

The earlier workers in dentistry have reason to complain of the writers of modern papers, that the results obtained by them are rarely quoted, although both ideas and results are plagiarized with as little show of conscience as that exhibited by the ordinary shoplifter. The older and more completely buried in some long since forgotten journal, the better for this class of writers, for then it is certain that discovery will be reduced to a minimum. There is another class that will dress up the old originals in modern scientific thought and technical expression, and the new dress gives a flavor of originality so striking that even the original writer would have difficulty in recognizing his own child.

The man with one paper sends it to a journal with a direct, or indirect, promise that this particular publication is the only one favored. The innocent editor, presuming that he is dealing with a truthful man, accepts it, and when issued he finds that every dental journal in the country has published it. This is thought to be a brilliant stroke of advertising genius, but the editor once imposed upon in this direction mentally makes a black-list in which the energetic individual has a prominent place, and he ceases to be a contributor to that periodical.

The man who reads the same paper to a half-dozen different State and inter-State societies, and finally winds up with the National Association, is the man the societies should especially learn to avoid. To the reader of journals there is nothing more annoying than to be obliged to sit and listen to a thrice-told tale and one without any remarkable originality. This is constantly being done and, doubtless, will have been repeated again at our annual conventions.

The man with one paper is very apt to consider that he is justified in culling from general and special knowledge to make a very full essay, illustrating the grains of wheat in a bushel of chaff. If this were all it might be pardoned, but he is very apt to give his own ideas upon subjects upon which he is manifestly ignorant. We have recently had several such instances. These would be amusing were it not for the fact that these crude notions tend to mislead others into false conceptions, and to accept stolid ignorance for authority. This fault is not confined to writers of papers, but is to be found in books written by careless authors. The writing upon any subject presupposes that he who attempts it has made himself master of all that has preceded his attempt, and in so far as he fails to do this he deceives his readers.

It is not pleasant to feel forced to call attention to the weaknesses of our co-workers, but the times require the truthful journalist and plain speaking. This is especially needed now, when the mental activity is more pronounced, than at any former period. The younger generation is making itself heard in unmistakable terms. The men trained in histology, chemistry, bacteriology, anatomy, etc., are coming rapidly to the fore with their original work. There is ample room for these, for the unsolved problems are many, not only here but in the more practical branches. There is no room, however, for the old stock subjects, nor is there room for the one paper in every journal in the land.

